# Decrease in community antibiotic consumption during the COVID-19 pandemic, EU/EEA, 2020

**DOI:** 10.2807/1560-7917.ES.2021.26.46.2101020

**Published:** 2021-11-18

**Authors:** Liselotte Diaz Högberg, Vera Vlahović-Palčevski, Cátia Pereira, Klaus Weist, Dominique L Monnet, ESAC-Net study group, ESAC-Net study group participants, Reinhild STRAUSS, Boudewijn CATRY, Ivan IVANOV, Marina PAYERL-PAL, Isavella KYRIAKIDOU, Berit MÜLLER-PEBODY, Janne SEPP, Emmi SARVIKIVI, Philippe CAVALIÉ, Birgitta SCHWEICKERT, Flora KONTOPIDOU, María MATUZ, Anna Margrét HALLDÓRSDÓTTIR, Ajay OZA, Filomena FORTINGUERRA, Ieva RUTKOVSKA, Rolanda VALINTELIENĖ, Stephanie SALEH, Peter ZARB, Stephanie NATSCH, Hege Salvesen BLIX, Anna OLCZAK-PIEŃKOWSKA, Ana SILVA, Gabriel Adrian POPESCU, Tomáš TESAŘ, Maja SUBELJ, Antonio LÓPEZ, Ragda OBEID

**Affiliations:** 1 European Centre for Disease Prevention and Control, Solna, Sweden; 2 Department of Clinical Pharmacology, University Hospital Rijeka, Rijeka/University of Rijeka Medical Faculty and Faculty of Health Studies, Rijeka, Croatia; 3 The members of the ESAC-Net study group are listed in the Investigators tab

**Keywords:** antibiotic consumption, surveillance, COVID-19

## Abstract

We present a European Union/European Economic Area-wide overview of the changes in consumption of antibacterials for systemic use (ATC J01) in the community between 2019 and 2020 as reported to the European Surveillance of Antimicrobial Consumption Network. Overall antibiotic consumption decreased by 18.3% between 2019 and 2020, the largest annual decrease in the network's two-decade history. We observed a strong association between the level of community antibiotic consumption in 2019 and the size of the decrease between 2019 and 2020.

The ongoing coronavirus disease (COVID-19) pandemic has since its start in early 2020 affected societies worldwide. In addition to the direct COVID-19-related morbidity and mortality, secondary effects on general communicable disease epidemiology and healthcare system delivery have also been reported [1,2].

Here, we present a European Union (EU)/European Economic Area (EEA)-wide overview of the changes in antibiotic consumption in the community in 2020 compared with previous years. We analysed the consumption of antibacterials for systemic use (ATC group J01), and focused on the community, i.e. the primary care sector, as this is where the changes between 2019 and 2020 were the largest at EU/EEA level and the most consistent among EU/EEA countries [3]. 

## EU/EEA population-weighted mean antibiotic consumption

**Figure 1:**
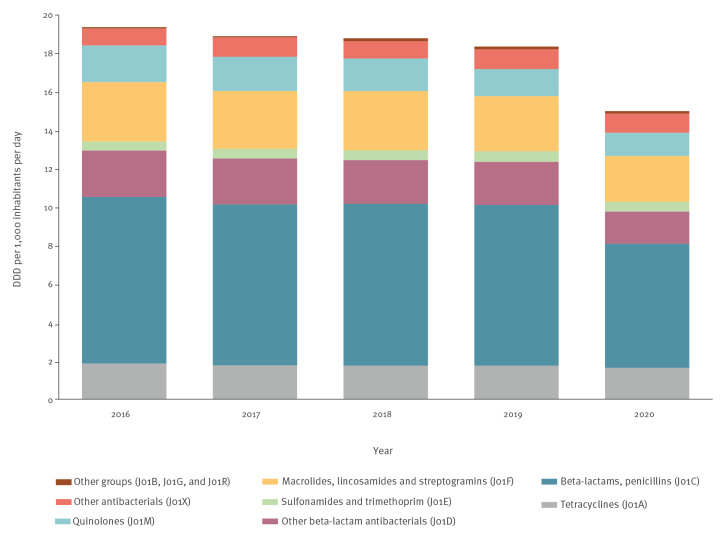
Consumption of antibacterials for systemic use (ATC group J01) in the community, population-weighted mean, by ATC group, 29 EU/EEA countries, 2016–2020

ATC: anatomical therapeutic chemical index; DDD: defined daily doses; EEA: European Economic Area; EU: European Union.

Our analyses are based on data reported to the European Surveillance of Antimicrobial Consumption Network (ESAC-Net), collected using a methodology described elsewhere [4] and expressed as defined daily doses (DDD) per 1,000 inhabitants per day, using the Anatomical Therapeutic Chemical (ATC) Index for 2021 [5]. Statistical analyses were performed with Stata version 16.0 [6]. The EU/EEA population-weighted mean is based on data from 29 countries. For 27 of these countries, separate data for the community sector were available and for two countries, data were imputed based on total consumption. The difference in antibiotic consumption between 2019 and 2020 was considerably larger than in previous years. While the EU/EEA population-weighted mean annual change in the consumption of antibacterials for systemic use (ATC group J01) during the period 2016 and 2019 was −0.34 DDD/1,000 inhabitants/day, representing a 1.8% annual decrease (median: −0.44 DDD/1,000 inhabitants/day or a 2.3% annual decrease), it decreased by −3.35 DDD/1000 inhabitants/day between 2019 and 2020, representing a 18.3% decrease (Figure 1).

The population-weighted mean is based on antimicrobial consumption data reported by 29 EU/EEA countries (Austria, Belgium, Bulgaria, Croatia, Cyprus, Czechia, Denmark, Estonia, Finland, France, Germany, Greece, Hungary, Iceland, Ireland, Italy, Latvia, Lithuania, Luxembourg, Malta, the Netherlands, Norway, Poland, Portugal, Romania, Slovakia, Slovenia, Spain and Sweden). Cyprus and Czechia only reported total care data (community and hospital consumption combined), and community consumption was imputed by assuming that community consumption contributed 90% of total care consumption.

**Figure 2:**
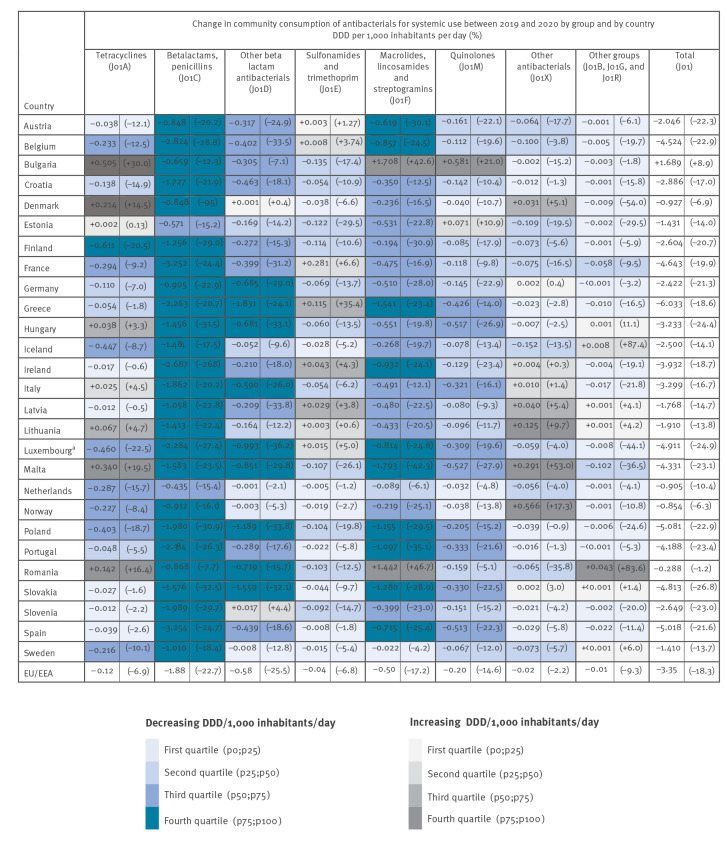
Consumption of antibacterials for systemic use (ATC group J01) in the community, EU/EEA countries, change between 2019 and 2020

ATC: anatomical therapeutic chemical index; DDD: defined daily doses; ECDC: European Centre for Disease Prevention and Control; EEA: European Economic Area; EU: European Union; TESSy: The European Surveillance System.

^a^ Luxembourg changed the data process between 2019 and 2020, which could impact comparability between the years.

For individual groups of antibiotics, the largest decrease in terms of DDD/1,000 inhabitants/day between 2019 and 2020 was observed for penicillins (ATC group J01C; −1.88 DDD/1000 inhabitants/day; −22.7%), followed by other beta-lactam antibacterials (J01D; −0.58 DDD/1,000 inhabitants/day; −25.5%), macrolides, lincosamides and streptogramins (J01F; −0.50 DDD/1,000 inhabitants/day; −17.2%), quinolones (J01M; −0.20 DDD/1,000 inhabitants/day; −14.6%), tetracyclines (J01A; −0.12 DDD/1,000 inhabitants/day; −6.9%), sulfonamides and trimethoprim (J01E; −0.04 DDD/1,000 inhabitants/day; −6.8%), other antibacterials (J01X; −0.02 DDD/1,000 inhabitants/day; −2.2%), and ATC groups J01B, J01G and J01R combined ; −0.01 DDD/1,000 inhabitants/day; −9.3%) (Figure 2).

Individual country analyses were available for 27 countries. EU/EEA refers to the corresponding population-weighted mean consumption based on antimicrobial consumption data reported by 29 EU/EEA countries (Austria, Belgium, Bulgaria, Croatia, Cyprus, Czechia, Denmark, Estonia, Finland, France, Germany, Greece, Hungary, Iceland, Ireland, Italy, Latvia, Lithuania, Luxembourg, Malta, the Netherlands, Norway, Poland, Portugal, Romania, Slovakia, Slovenia, Spain and Sweden). Cyprus and Czechia only reported total care data (community and hospital consumption combined),and their contribution to the EU/EEA population-weighted mean community consumption was imputed by assuming that community consumption contributed 90% of total care consumption Data extracted from ECDC TESSy on 7 September 2021.

## Country-level antibiotic consumption

Twenty-seven countries reported separate data on community consumption for 2019 and 2020. Of these, 26 reported a decrease in community consumption of antibacterials for systemic use in 2020 compared with 2019, while one country (Bulgaria) reported an increase during the same period (Figure 2).

**Figure 3:**
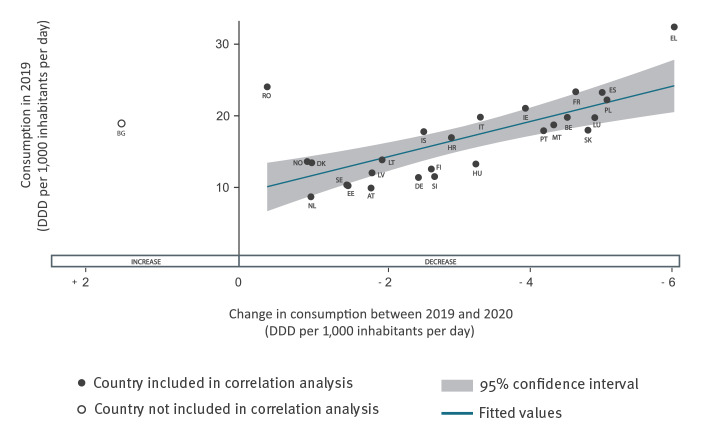
Scatter plot of consumption of antibacterials for systemic use (ATC group J01) in the community in 2019 vs change between 2019 and 2020, 27 EU/EEA countries

ATC: anatomical therapeutic chemical index; DDD: defined daily doses; ECDC: European Centre for Disease Prevention and Control; EEA: European Economic Area; EU: European Union; TESSy: The European Surveillance System.

Among the 26 countries reporting a decrease, there was a positive association between the level of antibiotic consumption in 2019 and the size of the decrease in antibiotic consumption between 2019 and 2020 measured in DDD/1,000 inhabitants/day (Pearson's correlation coefficient: 0.70, p value 0.0001); the decrease was proportionally larger in countries with high community antibiotic consumption than in countries with low antibiotic consumption (Figure 3).

Each dot represents a country. Included countries: Austria (AT), Belgium (BE), Bulgaria (BG), Croatia (HR), Denmark (DK), Estonia (EE), Finland (FI), France (FR), Germany (DE), Greece (EL), Hungary (HU), Iceland (IS), Ireland (IE), Italy (IT), Latvia (LV), Lithuania (LT), Luxembourg (LU), Malta (MT), the Netherlands (NL), Norway (NO), Poland (PL), Portugal (PT), Romania (RO), Slovakia (SK), Slovenia (SI), Spain (ES) and Sweden (SE). Countries which did not report data separately for the community sector, i.e. Cyprus and Czechia, were excluded from the analyses. Data extracted from ECDC TESSy on 7 September 2021.

Pearson's correlation coefficient: 0.70, p value 0.0001. Sensitivity analyses excluding two outliers, Greece and Romania: Pearson's correlation coefficient: 0.86, p value < 0.0001. Bulgaria was excluded from the correlation analysis as community consumption of antibacterials for systemic use consumption increased between 2019 and 2020.

For individual groups of antibiotics, there were various patterns of change in the 26 countries that reported a decrease overall. All 26 countries reported a decrease in the consumption of penicillins (ATC J01C) between 2019 and 2020, which corresponded to the largest reduction when expressed in DDD/1,000 inhabitants/day. A majority of countries also reported large reductions in the consumption of other beta-lactams, which includes cephalosporins (J01D), and of macrolides, lincosamides and streptogramins (J01F) (Figure 2). Only two countries (Bulgaria and Romania) reported an increase in the consumption of macrolides, lincosamides and streptogramins (J01F), and the increase in these countries (+1.71 DDD/1,000 inhabitants/day or a +42.6% increase, and +1.44 DDD/1,000 inhabitants/day or a + 46.7% increase, respectively) was the largest observed for any antibiotic group and country. For both countries, the increase in this group resulted from an increase in the consumption of azithromycin (J01FA10; +1.61 DDD/1,000 inhabitants/day or a +105.2% increase, and +1.92 DDD/1,000 inhabitants/day or a +178.2% increase, respectively). Five other countries also reported increased consumption of azithromycin during the same period, but at a much smaller magnitude (range: +0.01 to +0.26 DDD/1,000 inhabitants/day) and with an overall decrease in the consumption for the ATC group J01F.

## Discussion

The unprecedented decrease in community antibiotic consumption noted in the EU/EEA between 2019 and 2020 is the largest in ESAC-Net’s two-decade long antimicrobial consumption surveillance history, and one example of the far-reaching consequences of the COVID-19 pandemic. Although similar changes in community antibiotic consumption have been described at the local and national levels [7-10], this is the first report showing a substantial decrease across nearly all EU/EEA countries.

Because patient-level data are lacking in ESAC-Net, we could not assess whether the observed decrease reflects concomitant changes in disease transmission, healthcare utilisation patterns or prescription practices. Nevertheless, the large decrease noted for antibiotics commonly used to treat respiratory tract infections, e.g. penicillins and other beta-lactam antibacterials, is in line with the reported low incidence of non-COVID-19-related respiratory tract infections in the EU/EEA in 2020 [1,11]. This has been attributed to the non-pharmaceutical interventions put in place as a response to the pandemic, including physical distancing, respiratory etiquette, face masks and promotion of hand hygiene [12]. In addition, the decrease in community antibiotic consumption may have resulted from a decrease in the number of primary care consultations that were due to community lockdowns and reduced access to primary care during the period [2,8,9], which would most probably mainly result in fewer antibiotic prescriptions for mild and self-limiting infections. However, there was a more than 20-fold inter-country variation in the size of the decrease in community antibiotic consumption between 2019 and 2020. In general, countries with comparatively high community antibiotic consumption and showing a poor performance for quality indicators for antibiotic consumption in the community [13], reported the largest decreases between 2019 and 2020.

Inversely, two countries with high community antibiotic consumption reported either an increase (Bulgaria) or a comparatively small decrease (Romania) between 2019 and 2020. This was mainly explained by a large increase in the community consumption of macrolides, and specifically azithromycin. Azithromycin has been postulated to have antiviral and anti-inflammatory activity and has been studied for the treatment of COVID-19, however, multiple studies did not identify any clinical benefit [14].

It cannot be excluded that the ongoing COVID-19 pandemic could have impacted the quality of the data. However, with the exception of Luxembourg that implemented a change in data processing in 2020, we are not aware of any other changes in data sources or data collection processes between 2019 and 2020. 

Evidence-based international guidelines have discouraged antibiotics for prophylaxis or treatment in patients with mild or moderate COVID-19 infection without a suspicion of a bacterial co-infection [15,16], which would apply to most of the patients cared for in the community. Despite this, increased antibiotic consumption and indications of overuse in the community have been reported during the COVID-19 pandemic [17]. Our study shows that, with a few exceptions, this seems to have been less of an issue in the EU/EEA. It could be speculated whether one of the reasons behind this favourable situation is the coordinated and EU-wide ongoing effort towards prudent use of antimicrobials, an essential component of the European One Health Action Plan against Antimicrobial Resistance (AMR) [18]. However, since this study focused on community antibiotic consumption, the interpretation cannot be extrapolated to the hospital sector where the antibiotic consumption situation was more diverse [3].

## Conclusion

The large changes in antibiotic consumption concomitant with the COVID-19 pandemic need to be further evaluated, both in countries with high and low antibiotic consumption, and areas of inappropriate antibiotic use need to be addressed within antimicrobial stewardship programmes in outpatient settings. It is still unclear whether this reduced community antibiotic consumption was sustained throughout 2021 and what implications it may have on antibiotic resistance. Robust surveillance systems will continue to be vital to monitor the situation.
